# Pharmacological inhibition of IL-6 trans-signaling improves compromised fracture healing after severe trauma

**DOI:** 10.1007/s00210-018-1483-7

**Published:** 2018-03-01

**Authors:** Kathrin Kaiser, Katja Prystaz, Anna Vikman, Melanie Haffner-Luntzer, Stephanie Bergdolt, Gudrun Strauss, Georg H. Waetzig, Stefan Rose-John, Anita Ignatius

**Affiliations:** 1 Institute of Orthopedic Research and Biomechanics, Trauma Research Center Ulm, University Medical Center Ulm, 89081 Ulm, Germany; 2 Department of Pediatrics and Adolescent Medicine, University Medical Center Ulm, 89075 Ulm, Germany; 3 CONARIS Research Institute AG, 24118 Kiel, Germany; 4 Institute of Biochemistry, Christian-Albrechts-University of Kiel, 24118 Kiel, Germany

**Keywords:** Trauma, Bone fracture healing, IL-6, Inflammation, Classic signaling, Trans-signaling

## Abstract

Patients with multiple injuries frequently suffer bone fractures and are at high risk to develop fracture healing complications. Because of its key role both in systemic posttraumatic inflammation and fracture healing, the pleiotropic cytokine interleukin-6 (IL-6) may be involved in the pathomechanisms of trauma-induced compromised fracture healing. IL-6 signals are transmitted by two different mechanisms: classic signaling via the membrane-bound receptor (mIL-6R) and trans-signaling via its soluble form (sIL-6R). Herein, we investigated whether IL-6 classic and trans-signaling play different roles in bone regeneration after severe injury. Twelve-week-old C57BL/6J mice underwent combined femur osteotomy and thoracic trauma. To study the function of IL-6, either an anti-IL-6 antibody, which inhibits both IL-6 classic and trans-signaling, or a soluble glycoprotein 130 fusion protein (sgp130Fc), which selectively blocks trans-signaling, were injected 30 min and 48 h after surgery. Bone healing was assessed using cytokine analyses, flow cytometry, histology, micro-computed tomography, and biomechanical testing. Selective inhibition of IL-6 trans-signaling significantly improved the fracture healing outcome after combined injury, as confirmed by accelerated cartilage-to-bone transformation, enhanced bony bridging of the fracture gap and improved mechanical callus properties. In contrast, global IL-6 inhibition did not affect compromised fracture healing. These data suggest that classic signaling may mediate beneficial effects on bone repair after severe injury. Selective inhibition of IL-6 trans-signaling might have therapeutic potential to treat fracture healing complications in patients with concomitant injuries.

**Electronic supplementary material:**

The online version of this article (10.1007/s00210-018-1483-7) contains supplementary material, which is available to authorized users.

## Introduction

Patients with multiple injuries frequently suffer from bone fractures and are at high risk to develop fracture healing complications, including non-unions (Bhandari et al. 2003; Karladani et al. 2001; Zura et al. 2016). One reason for poor bone regeneration after severe trauma is the overwhelming systemic posttraumatic inflammation, which is triggered by endogenous alarm signals, including cell and matrix debris, being released from the injured tissues (Bastian et al. 2011; Claes et al. 2012; Pape et al. 2010). The immune response is accompanied by a flood of inflammatory mediators, among them the pleiotropic cytokine interleukin-6 (IL-6) (Lenz et al. 2007; Lord et al. 2014; van Griensven 2014; Volpin et al. 2014). IL-6 is considered to be a key mediator in this complex scenario because of its significant correlation with injury severity and clinical complications (Alper et al. 2016; Cuschieri et al. 2010; Frink et al. 2009; Gebhard et al. 2000). It provokes both pro-inflammatory and protective effects. IL-6 is pivotal for the amplification of the inflammatory signal by stimulating leukocyte recruitment and the production of other inflammatory mediators. Furthermore, it induces the acute-phase response, activates the complement and coagulation cascades, and increases hematopoiesis, thrombocytosis, and vascular permeability (Tanaka et al. 2016). IL-6 also contributes to the resolution of inflammation, for example, by inducing the shift from pro-inflammatory M1 to anti-inflammatory M2 macrophages (Mauer et al. 2014) and the recruitment of mesenchymal stem cells from their niches (Loi et al. 2016).

Because of its key role in posttraumatic inflammation, we hypothesize that IL-6 might crucially contribute to compromised fracture healing in patients with concomitant injuries. Supporting this, bone healing complications are observed more frequently in patients with, for example, osteoporosis and rheumatoid arthritis, which are associated with an inflammatory phenotype that includes increased IL-6 levels (Edwards and Williams 2010; Hardy and Cooper 2009; Oei et al. 2015). Moreover, IL-6 exerts crucial regulatory functions in all stages of bone repair (Ai-Aql et al. 2008; Kon et al. 2001; Wallace et al. 2011; Yang et al. 2007). In the fracture hematoma, IL-6 controls the recruitment and activity of immune cells and angiogenesis (Ai-Aql et al. 2008; Prystaz et al. 2017; Walters et al. 2017). In the repair phase, IL-6 regulates intramembranous and endochondral ossification and fracture callus remodeling (Wallace et al. 2011; Yang et al. 2007).

Essentially, IL-6 signals are transmitted by two distinct mechanisms: In IL-6 classic signaling, IL-6 activates its membrane-bound receptor (mIL-6R), which is mainly expressed by some immune cells and hepatocytes. In IL-6 trans-signaling, IL-6 binds to its soluble receptor (sIL-6R), which is preferentially released from leukocytes following proteolytic cleavage of the mIL-6R, particularly in response to an inflammatory stimulus (McFarland-Mancini et al. 2010; Yan et al. 2016). In both pathways, intracellular signal transduction is induced after the association of the IL-6/IL-6R complexes with the ubiquitously expressed transmembrane glycoprotein 130 (gp130) (Rose-John 2012; Scheller et al. 2014). Whereas IL-6 classic signaling is regarded to regulate homeostasis and support regeneration, IL-6 trans-signaling rather acts as a danger signal driving inflammation (Barkhausen et al. 2011; Rose-John 2012; Scheller et al. 2011; Zhang et al. 2013). In a recent study, we deciphered IL-6 actions in bone healing by discriminating between both signaling pathways, using a mouse model of isolated fracture healing, which induces only mild systemic inflammation and leads to uneventful bone repair (Prystaz et al. 2017). We demonstrated that the classic IL-6 pathway is important for a balanced systemic and local immune response after fracture by regulating the production of acute-phase proteins in the liver, the recruitment of immune cells to the fracture hematoma, and by inducing regenerative downstream processes augmenting bone repair. By contrast, IL-6 trans-signaling plays only a subordinate role in uncomplicated fracture healing (Prystaz et al. 2017). Because severe trauma is associated with increased levels of IL-6 (Alper et al. 2016; Cuschieri et al. 2010; Frink et al. 2009; Gebhard et al. 2000) and its soluble receptor (Kleber et al. 2015) and trans-signaling is proposed to account for many deleterious effects of IL-6 (Barkhausen et al. 2011), the present study investigated the hypothesis that IL-6 trans-signaling is involved in the pathomechanisms of trauma-induced impaired bone healing. We used a mouse model of combined femur fracture and thoracic trauma, which provokes systemic inflammation and compromised bone repair, mimicking the clinical situation of fracture patients with multiple injuries (Kemmler et al. 2015; Kovtun et al. 2016). To determine the role of IL-6, we blocked either global IL-6 signaling, using an anti-IL-6 antibody, or IL-6 trans-signaling, using sgp130Fc, an artificial fusion protein, which neutralizes the IL-6/sIL-6R-complex (Jostock et al. 2001; Rose-John et al. 2007). Our results revealed that inhibition of IL-6 trans-signaling significantly improved bone healing after severe trauma, whereas global IL-6 inhibition had no effect. This may have therapeutic implications for patients with fracture healing complications.

## Material and methods

### Study design

All experiments were performed in compliance with the international regulations for the care and use of laboratory animals (Directive 2010/63/EU) and with the approval of the local ethical committee (Regierungspräsidium Tübingen, Reg. No. 1166 and 1247). Male 12-week-old C57BL/6 J mice were purchased from Charles River (Sulzfeld, Germany). The mice were maintained in groups of two to five animals per cage (370 cm^2^) on a 14 h light and 10 h dark circadian rhythm with water and food ad libitum.

To analyze fracture healing, we used a standardized osteotomy model (Röntgen et al. 2010). The osteotomy was combined with an additional thoracic trauma as described previously (Kemmler et al. 2015) to induce systemic inflammation. The mice were randomly assigned to the following groups (Table 1): (I) mice with isolated femur fracture (Fx); (II) mice with combined fracture and thoracic trauma (Fx + TxT); (III) mice with Fx + TxT, which received an anti-IL-6 antibody to block global IL-6 signaling; and (IV) mice with Fx + TxT which were treated with sgp130Fc, a selective inhibitor of trans-signaling. The animals were euthanized 3 h or 1, 10, and 21 days after surgery using isoflurane overdose and terminal cardiac puncture. To reduce the number of mice and address the 3Rs principles for ethical use of animals, vehicle-treated control mice with isolated fracture derived from a previous study (Prystaz et al. 2017) were included in the control group of the present study.

**Table 1:** Experimental groups and treatments

Group	Surgery	Treatment	Compound
I	Fx	Control	PBS
II	Fx + TxT	Control	PBS
III	Fx + TxT	IL-6 inhibition	Anti-IL-6
IV	Fx + TxT	IL-6 inhibition	sgp130Fc

*Fx* fracture, *TxT* thoracic trauma, *PBS* phosphate-buffered saline, *sgp130Fc* soluble glycoprotein 130-Fc fusion protein, *anti-IL-6* anti-IL-6 antibody

### Femur osteotomy and thoracic trauma

Mice were anesthetized with 2% isoflurane (Forene, Abbott, Wiesbaden, Germany). The femur osteotomy was described in detail before (Röntgen et al. 2010). Briefly, the right femur was exposed by penetrating the fascia latae between the gluteus superficialis and biceps femoris muscles. An external fixator (RISystem, Davos, Switzerland) was fitted to the femur in a cranio-lateral position with four mini-Schanz screws. An osteotomy gap was created using a 0.4-mm Gigli saw at the femur midshaft. Then, the muscles were sutured with absorbable (Vicryl®; J&J, Norderstedt, Germany), and the skin with nonabsorbable thread (Resolon®; Resorba, Nuernberg, Germany). For pain treatment, tramadol hydrochloride (Tramal, Gruenenthal GmbH, Aachen, Germany) was applied in the drinking water, starting 1 day prior to surgery until 3 days post-surgery. Immediately before surgery, mice were treated with a single dose of antibiotic (clindamycin-2-dihydrogen-phosphate, 45 mg/kg, Clinda-saar 600 mg, MIP Pharma GmbH, Blieskastel, Germany). The thoracic trauma was applied immediately after fracture, while the mice were still under general anesthesia (Kemmler et al. 2015; Knoferl et al. 2003). Briefly, the mice were fixed in a supine position. A single blast wave was applied on the middle of the thorax using a blast wave generator, which was centered 2 cm above the thorax. The blast wave generator consists of two parts; the upper part serves as an air pressure reservoir, whereas the lower nozzle is directed towards the animals’ chest. Between the two parts, there is a thin membrane that ruptures at a pressure of exactly 13 bar, leading to a single blast wave hitting the thorax. This induces a standardized bilateral, isolated lung contusion (Kemmler et al. 2015).

### Inhibition of IL-6 signaling

To selectively inhibit IL-6 trans-signaling, mice received 0.5 mg/kg sgp130Fc (CONARIS Research Institute AG, Kiel, Germany) 30 min and 48 h after osteotomy. Sgp130Fc is an artificial fusion protein of the extracellular domain of gp130 dimerized by the Fc domain of human immunoglobulin G1 (IgG1), which selectively binds to the IL6/sIL-6 complex (Jostock et al. 2001). For global IL-6 inhibition, 2.0 mg/kg of a neutralizing rat anti-murine anti-IL-6 antibody (anti-IL-6) (clone MP5-20F3, BD Biosciences, Heidelberg, Germany) were applied at the same time points (Barkhausen et al. 2011; Prystaz et al. 2017). Classic signaling cannot be inhibited specifically. However, its influence can be deduced by comparing the effects of IL-6 global and trans-signaling inhibition (Barkhausen et al. 2011). Control mice received phosphate-buffered saline solution (PBS, Fisher Scientific GmbH, Schwerte, Germany). IgG as a vehicle solution was tested in a previous study and showed now effects compared to PBS (Prystaz et al. 2017). All agents were injected intraperitoneally.

### Multiplex cytokine analysis and ELISA

To assess systemic posttraumatic inflammation, plasma and serum were obtained 3 h and 1 day after surgery. The fracture hematoma was harvested and lysed as described previously (Prystaz et al. 2017). The lungs were flushed with 500 μL of ice-cold PBS to investigate the pulmonary inflammation (Perl et al. 2006). Broncho-alveolar lavage (BAL) fluids were centrifuged at 300×*g* for 15 min and supernatants were stored at −80 °C for further analyses.

A mouse Multiplex Cytokine Kit (ProcartaPlex, eBioscience, Frankfurt, Germany) was used to quantify plasma, BAL, and hematoma concentrations of the pro-inflammatory cytokines IL-6, IL-1β, tumor necrosis factor-α (TNF-α), and interferon-γ (IFN-γ) as well as the anti-inflammatory mediators IL-10, IL-13, IL-4, and the chemokines monocyte chemotactic protein 1 (MCP-1), chemokine (C-X-C motif) ligand 1 (CXCL1), and macrophage inflammatory protein-1α (MIP-1α). Samples were analyzed using the Luminex® 100 Total System (Bio-Rad Laboratories, Hercules, USA). The total protein concentration of the hematoma samples was determined with the Pierce™ BCA Protein Assay Kit (Fisher Scientific GmbH) and the cytokine values were normalized to the measured protein concentration. The sIL-6R serum levels were determined using a mouse sIL-6R enzyme-linked immunosorbent assay (ELISA; R&D Systems, Minneapolis, USA). A Simplex Kit (CRP Mouse ProcartaPlex™ Simplex Kit, Invitrogen™ Carlsbad, USA) was used to determine C-reactive protein (CRP) levels in plasma samples 3 h and 1 day after surgery according to the manufacturer’s protocol and data were analyzed using the Luminex® system described above.

### Real-time PCR

Liver samples were prepared as described previously (Prystaz et al. 2017). In brief, the samples were stored in RNA*later*®-ICE Frozen Tissue Transition Solution (Fisher Scientific GmbH). They were homogenized using a disperser (Miccra®, Müllheim, Germany), incubated with 1 ml Trizol, 0.2 ml chloroform was added, and the samples were centrifuged at 12,000×*g* for 30 min. RNA was isolated using the PureLink® RNA Mini Kit (Fisher Scientific GmbH). Further processing and qPCR analysis were performed as previously described (Haffner-Luntzer et al. 2014; Prystaz et al. 2017). Glyceraldehyde 3-phosphate dehydrogenase (*Gapdh*) served as a housekeeping gene. The expression of chemokines and acute-phase proteins was measured using specific primers for *CXCL1*, serum amyloid A (*Saa*), and CRP (*Crp*) (Table 2). Relative gene expression was calculated using the ΔΔCt method with PCR efficiency correction using LinReg PCR 2015.3 (Academic Medical Centre, Amsterdam, Netherlands) (Ramakers et al. 2003). Cycle threshold (Ct) values obtained for each sample were normalized to those of the housekeeping gene *Gapdh* and the control group with isolated fracture.

**Table 2:** Primer sequences

Gene	Primer
*Cxcl1* (*CXXL1*)	F: 5′-TCT CCG TTA CTT GGG GAC AC-3′
	R: 5′-CCA CAC TCA AGA ATG GTC GC-3′
*Saa* (*SAA*)	F: 5′-GAC ACC AGG ATG AAG CTA CTC A-3′
	R: 5′-CTT GGA AAG CCT CGT GAA CA-3′
*Crp* (*CRP*)	F: 5′-ATC CCA GCA GCA TCC ATA GC-3′
	R: 5′-AAC ATG TCT TCA TGA CCA AAA GTC C-3′
*Gapdh* (*GAPDH*)	F: 5′-ACC CAG AAG ACT GTG GAT GG-3′
	R: 5′-GGA TGC AGG GAT GAT GTT CT-3′

Genes with protein names in parentheses

*CXCL1* chemokine (C-X-C motif) ligand 1, *SAA* serum amyloid A, *CRP* C-reactive protein, *GAPDH* glyceraldehyde-3-phosphate dehydrogenase

### Flow cytometry

Immune cell populations in the fracture hematoma were determined by flow cytometry. Hematoma samples were harvested and homogenized by passing them through a 70-μm cell strainer (Corning Inc., Durham, NC). The resulting cell suspension was stained for 30 min on ice with the following antibodies against the indicated surface markers: anti-Ly-6G-V450 antibody (No. 560603 BD Biosciences), anti-CD11b-Alexa Fluor 700 (No. 56-0112 eBioscience), anti-F4/80-FITC (No. 11-4801 eBioscience), anti-CD3e-PE-Cyanine7 (No. 25-0031 eBioscience), and anti-CD19-PE antibody (No. 12-0193 eBioscience). Corresponding isotype-matched controls from the respective manufacturers served as negative controls. Dead cells were excluded using 7-aminoactinomycin D (7AAD) staining (Sigma Aldrich, Taufkirchen, Germany). Live cells were gated for the following cell populations: neutrophil granulocytes (CD11b+, Ly-6G+), macrophages (CD11b+, Ly-6G−, F4/80+), B cells (CD3−, CD19+), and T cells (CD3+, CD19−). The samples were analyzed using a LSR II flow cytometer (BD Biosciences) and FlowJo software (10.0.8r1, FlowJo, Ashland, USA).

### Histomorphometry and immunohistochemistry

Lungs (3 h and 1 day) were harvested and fixed in 4% buffered formalin solution (Otto Fischar GmbH & Co. KG, Saarbruecken, Germany). They were embedded in paraffin and stained with hematoxylin and eosin (Mayer’s hemalum solution, Merck KGaA®, Darmstadt, Germany and Eosin Y, Applichem, Darmstadt, Germany) for morphological investigations. Neutrophil granulocytes were identified using a Ly-6G-antibody (1:300 LEAF™, No. 127632 BioLegend, Fell, Germany).

Fractured femurs (days 1 and 10) were fixed in 4% buffered formalin solution, decalcified in 20% ethylenediaminetetraacetic acid (EDTA) for 10–12 days, and embedded in paraffin for immunohistochemistry. Femur samples collected 21 days after surgery were embedded in methyl methacrylate (MMA) without decalcification. For tissue quantification, femur sections were stained with either Safranin-O (paraffin sections; Merck Chemicals GmbH, Darmstadt, Germany), which stains mainly cartilage, or Giemsa (MMA-embedded samples; AppliChem). The relative amounts of osseous, cartilage, and fibrous tissues were evaluated in the callus between the inner two pinholes using image analysis software (MMAF Version 1.4.0 MetaMorph®, Leica, Heerbrugg, Switzerland). For immunostaining, we used the following antibodies and dilutions: neutrophil granulocytes 1:300 LEAF™ anti-mouse Ly-6G antibody (No. 127632 BioLegend), macrophages 1:500 rat anti-mouse F4/80 antibody (No. MCA497GA AbD Serotec, Puchheim, Germany), and collagen X 1:200 rabbit anti-mouse collagen X antibody (No. ABIN1077945 Antibodies-Online, Atlanta, USA). Secondary antibodies and dilutions: 1:200 goat anti-rabbit IgG secondary antibody (No. B2770 Life Technologies, Carlsbad, USA) and 1:200 goat anti-rat IgG secondary antibody (No. A10517 Life Technologies). Species-specific IgG subtype mixtures obtained from the respective manufactures were used as negative controls. For signal detection, Vectastain Elite ABC kit and Vector NovaRED substrate (both Vector laboratories Inc., Burlingame, USA) were applied according to the manufacturer’s protocols. Sections were counterstained with hematoxylin (Waldeck, Münster, Germany) and analyzed by light microscopy (Leica DMI6000B, Leica). The relative proportion of collagen X-positive stained cartilage was evaluated in the fracture callus between the inner two pinholes using the image analysis software described above.

### Biomechanical testing

To assess the bending stiffness of the fractured femurs explanted on day 21, a non-destructive three-point bending test was performed (Röntgen et al. 2010). Briefly, after removal of the external fixator, an axial load with a maximum of 2 N was applied to the top of the cranio-lateral callus side using a materials testing machine (1454, Zwick GmbH & Co KG, Ulm, Germany). The bending stiffness was calculated from the slope of the load-deflection curve (Röntgen et al. 2010).

### Micro-computed tomography

After biomechanical testing, the fractured femurs were scanned using a micro-computed tomography (μCT) scanning device (Skyscan 1172; Bruker, Kontich, Belgium) operating at a voxel resolution of 8 μm (50 kV, 200 mA) to evaluate bone formation and structural parameters of the fracture callus. Phantoms with a defined hydroxyapatite density (250 and 750 mg/cm^3^) were used to calibrate and assess the bone mineral density. The volume of interest comprised the periosteal callus between the two inner pinholes and the fracture gap. A global threshold of 642 mg hydroxyapatite/cm^3^ was applied to discriminate between mineralized and non-mineralized tissues (Morgan et al. 2009) according to the American Society for Bone and Mineral Research guidelines for μCT (Bouxsein et al. 2010).

### Statistical analysis

All data are presented as the mean ± standard deviation. Statistical analysis was performed using GraphPad Prism 6 (GraphPad Software, La Jolla, USA). Data were tested for normal distribution with Shapiro-Wilk test and then compared by either Kruskall-Wallis and Dunn’s post hoc test or by one-way analysis of variation and Fishers LSD post hoc test. The level of significance was set at *p* ≤ 0.05. The main outcome parameter of flexural rigidity of the fractured femur (power: 80%, *α* = 0.05) obtained from previous studies (Kovtun et al. 2016) was used to calculate sample size, which is indicated in the figure legends.

## Results

### Global IL-6 inhibition does not influence compromised bone repair induced by combined fracture and thoracic trauma

**Fig. 1:**
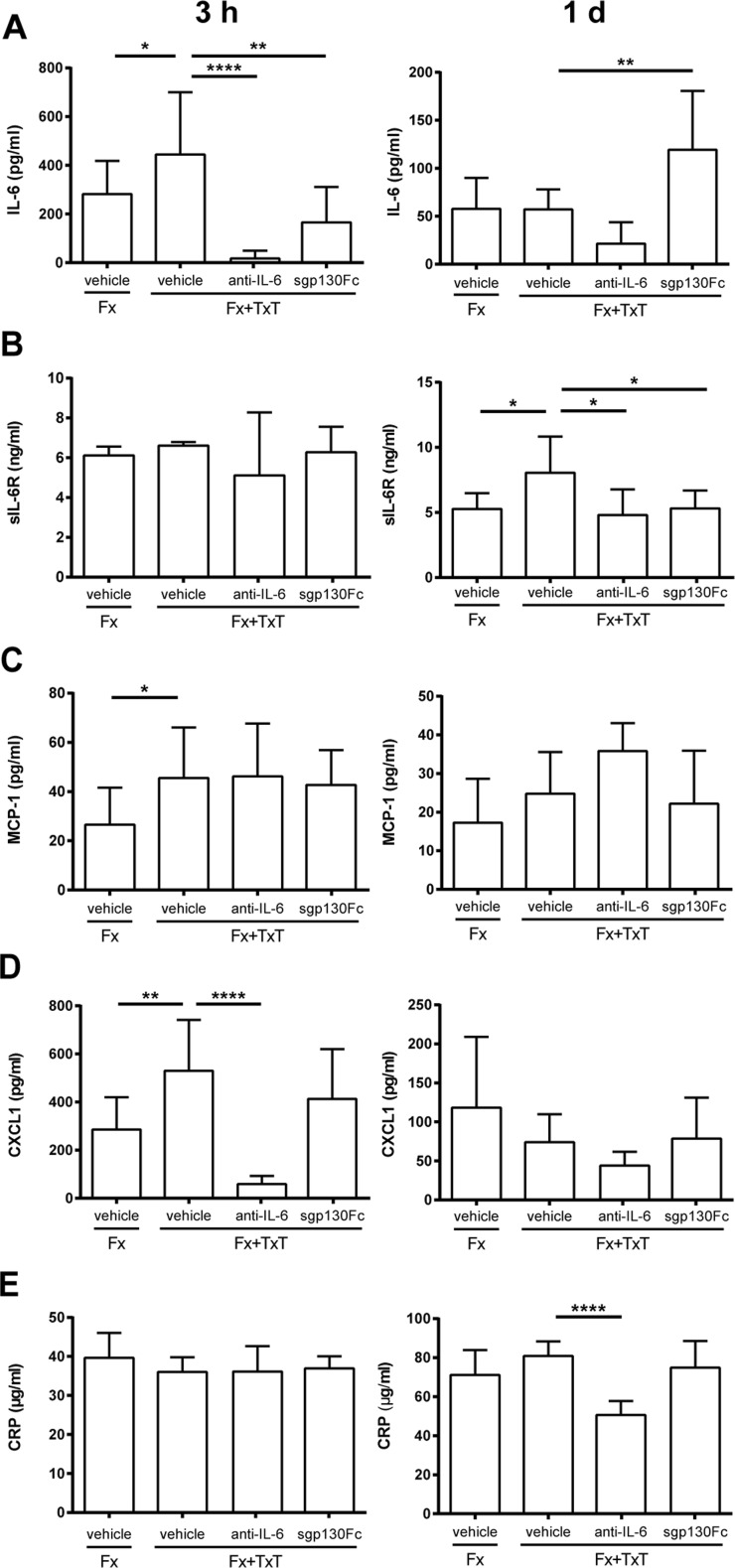
Inflammatory mediators in the blood 3 h and 1 day after fracture (Fx) and combined fracture and thoracic trauma (Fx + TxT) in vehicle-, anti-IL-6 antibody-, and sgp130Fc-treated mice. Data are displayed as means ± standard deviation. **a** *n* = 5–10; **b** *n* = 5–6; **c**, **d** *n* = 6–10; **e** *n* = 6. *p ≤ 0.05; **p ≤ 0.01; ****p ≤ 0.0001. Data of untreated animals are presented in Supplemental Table 1

**Fig. 2:**
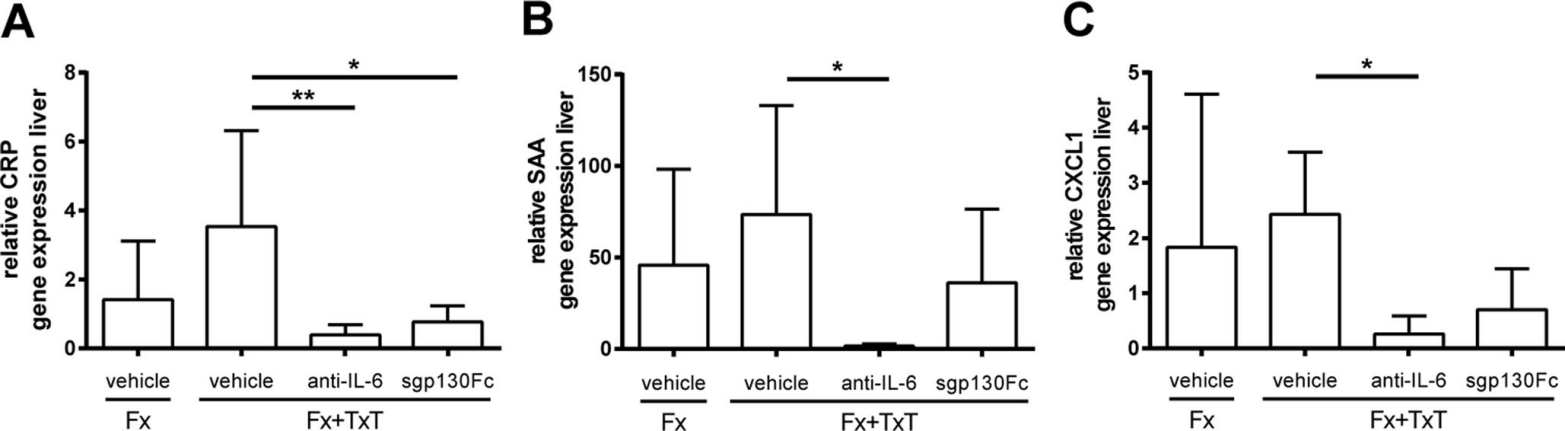
Hepatic acute-phase reaction 1 day after fracture (Fx) and combined fracture and thoracic trauma (Fx + TxT) in vehicle-, anti-IL-6- antibody-, and sgp130Fc-treated mice. Relative gene expression of **a** *CRP* C-reactive protein, **b** *SAA* serum amyloid A, and **c** *CXCL1* chemokine (C-X-C motif) ligand 1 in the liver. Data are displayed as means ± standard deviation. *n* = 4–5. *p ≤ 0.05; **p ≤ 0.01

**Fig. 3:**
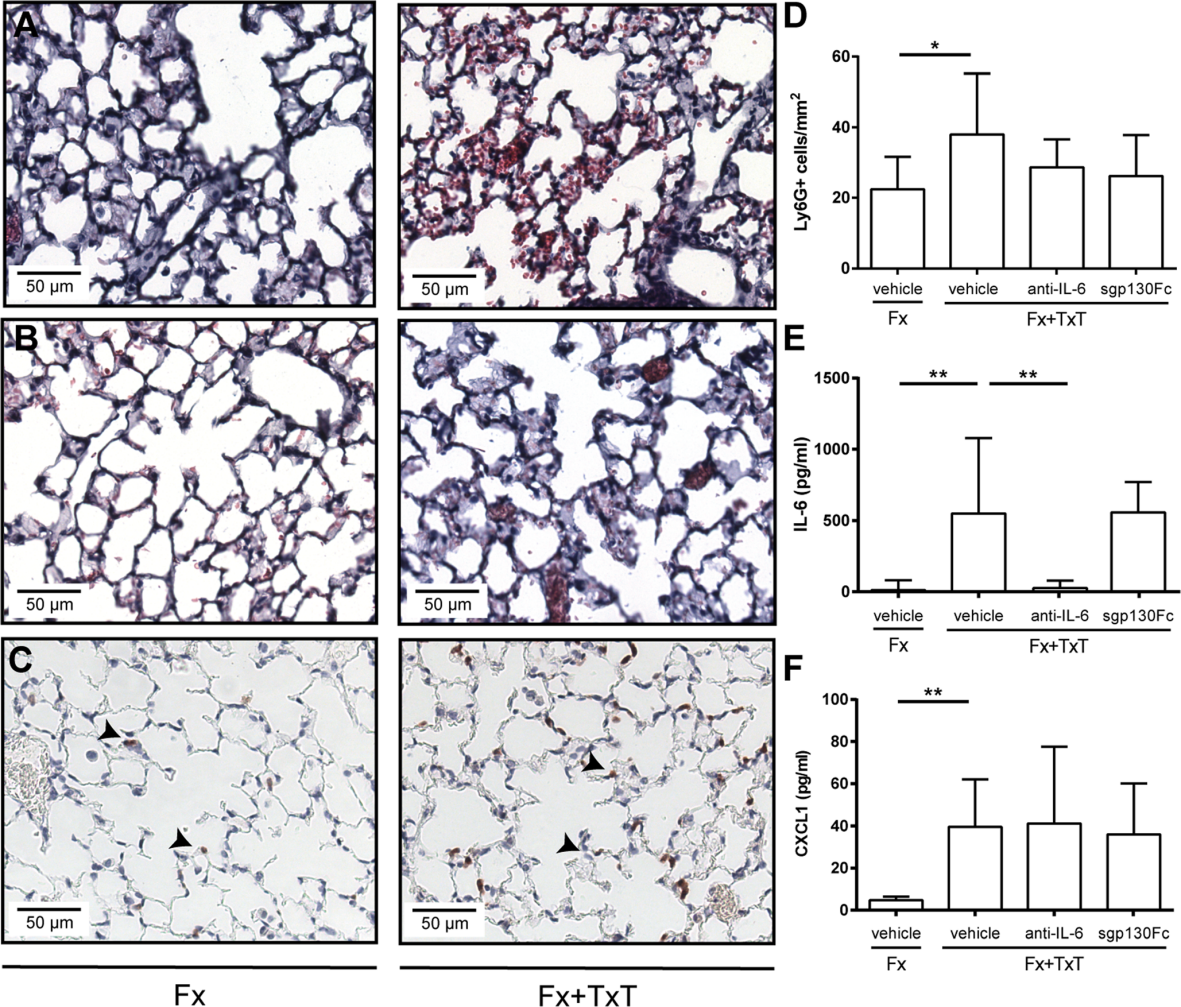
Pulmonary inflammation 3 h and 1 day after fracture (Fx) and combined fracture and thoracic trauma (Fx + TxT) in vehicle-, anti-IL-6 antibody-, and sgp130Fc-treated mice. **a** Representative images of hematoxylin and eosin (H&E) stained lungs of vehicle-treated mice after 3 h and **b** 1 day. **c** Representative images of lungs stained for neutrophils (Ly-6G+); arrowheads indicate positively stained cells. **d** Neutrophil (Ly-6G+) number in lung tissue. **e** IL-6 and **f** *CXCL1* chemokine (C-X-C motif) ligand 1 concentrations in the broncho-alveolar lavage fluid after 3 h. Data are displayed as means ± standard deviation. **D** *n* = 5–6; **E**, **F** *n* = 8–9. *p ≤ 0.05; **p ≤ 0.01. Data of untreated animals are presented in Supplemental Table 1

**Fig. 4:**
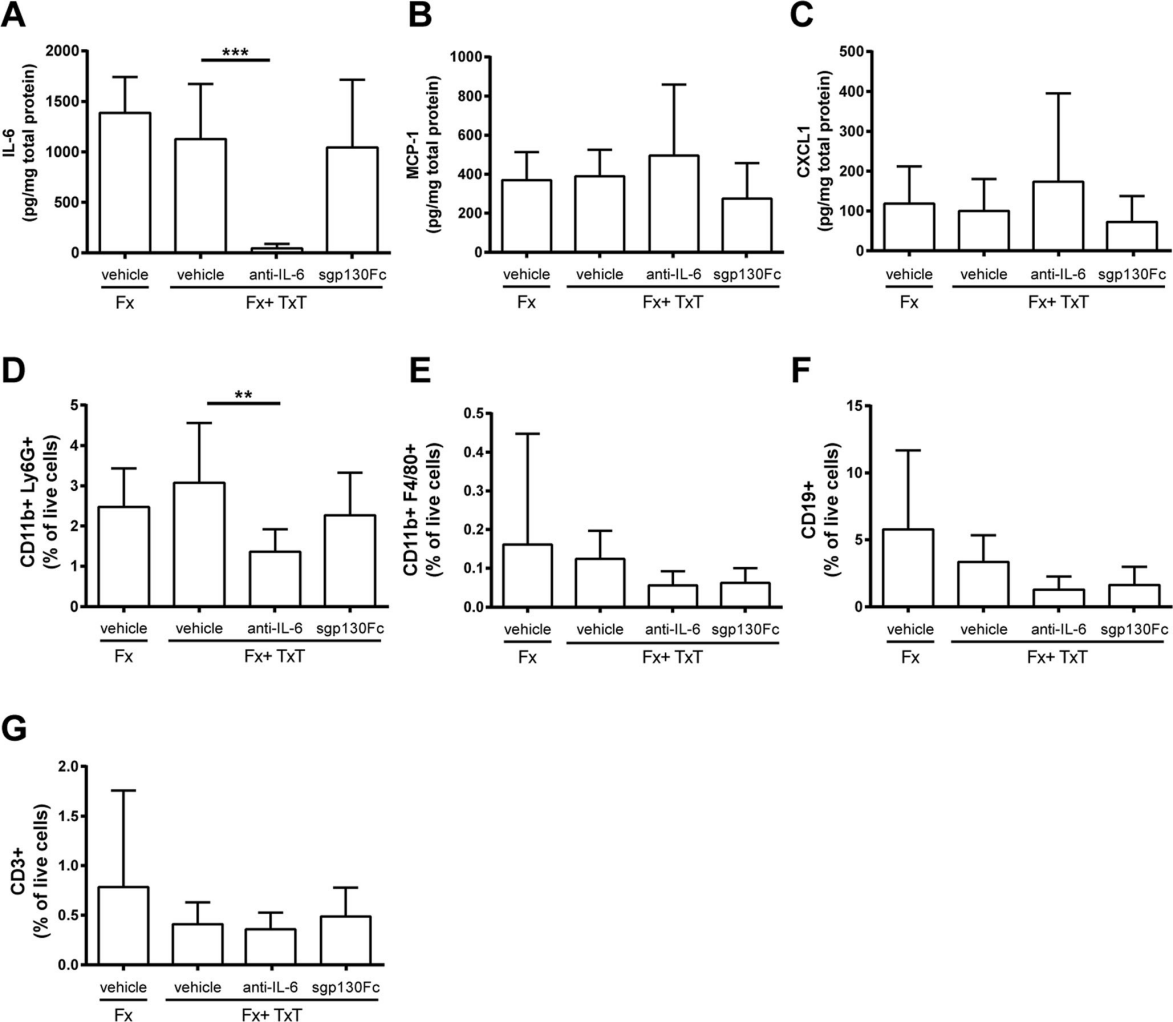
Inflammatory mediators and immune cells in the fracture hematoma 3 h and 1 day after fracture (Fx) and combined fracture and thoracic trauma (Fx + TxT) in vehicle-, anti-IL-6 antibody-, and sgp130Fc-treated mice. Data are displayed as means ± standard deviation. **a** IL-6, **b** *MCP-1* monocyte chemotactic protein 1, and **c** *CXCL1* chemokine (C-X-C motif) ligand 1 concentrations after 3 h. **d** Proportion of neutrophils (CD11b+, Ly6G+), **e** macrophages (CD11b+, F4/80+), **f** B cells (CD19+), and **g** T cells (CD3+). **a**–**c** *n* = 6–7, **d** *n* = 6–8, **e**–**g** *n* = 7–8. **p ≤ 0.01, ***p ≤ 0.001

**Fig. 5:**
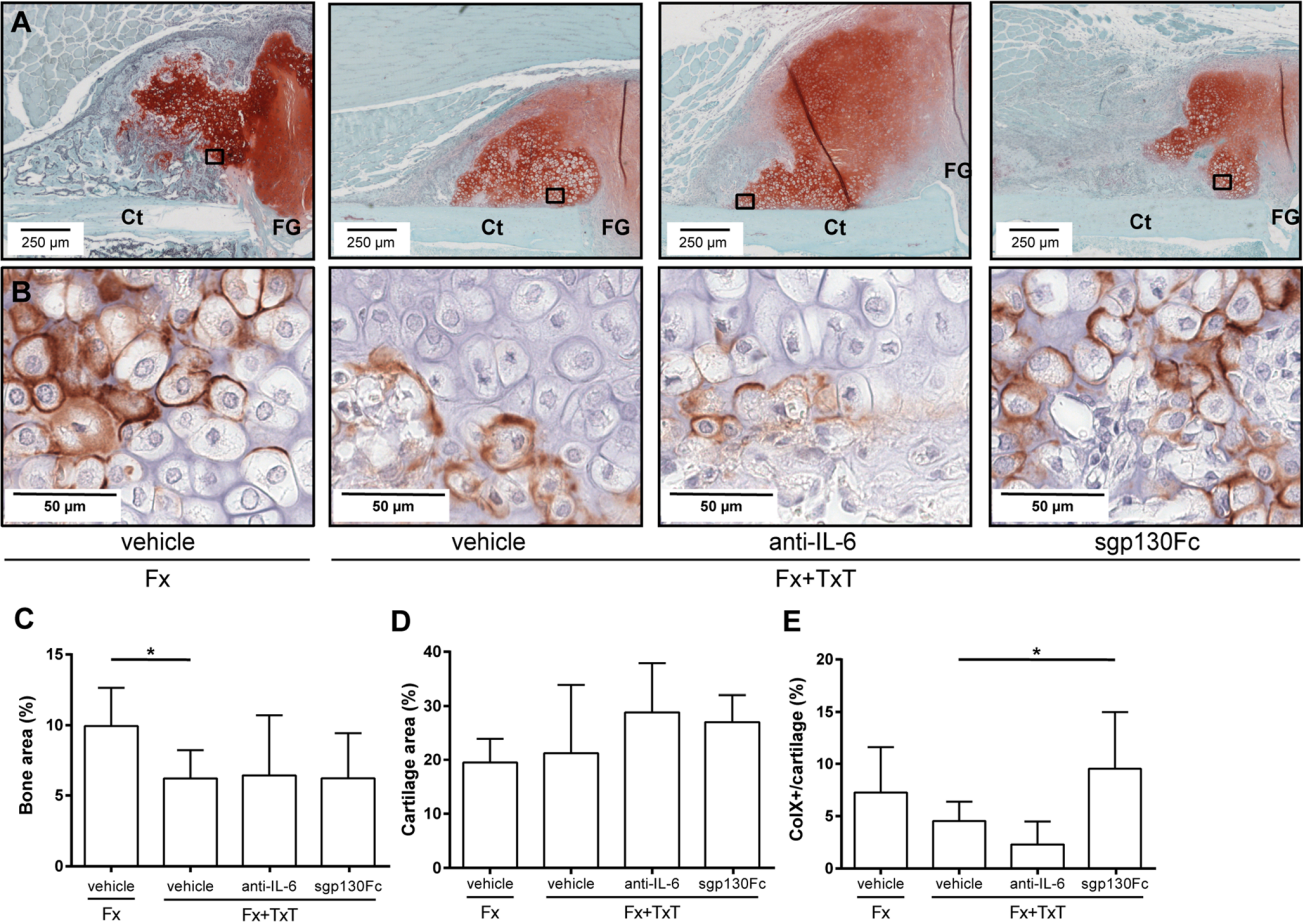
Histomorphometrical analyses of the fracture callus on day 10 after fracture (Fx) and combined fracture and thoracic trauma (Fx + TxT) in vehicle-, anti-IL-6 antibody-, and sgp130Fc-treated mice. **a** Representative histological images of the fracture callus stained with Safranin-O: *Ct* cortex, *FG* fracture gap. Boxed areas in **a** indicate the location of the higher magnified images in **b**. **b** Immunostaining of collagen type X. **c** Relative amount of bone and **d** cartilage in the fracture callus. **e** Proportion of collagen type X (ColX)-positive stained cartilage of the total cartilage determined by immunohistochemistry. Data are displayed as mean ± standard deviation. **c**, **d** *n* = 6; (**e**) *n* = 4–5. *p ≤ 0.05

**Fig. 6:**
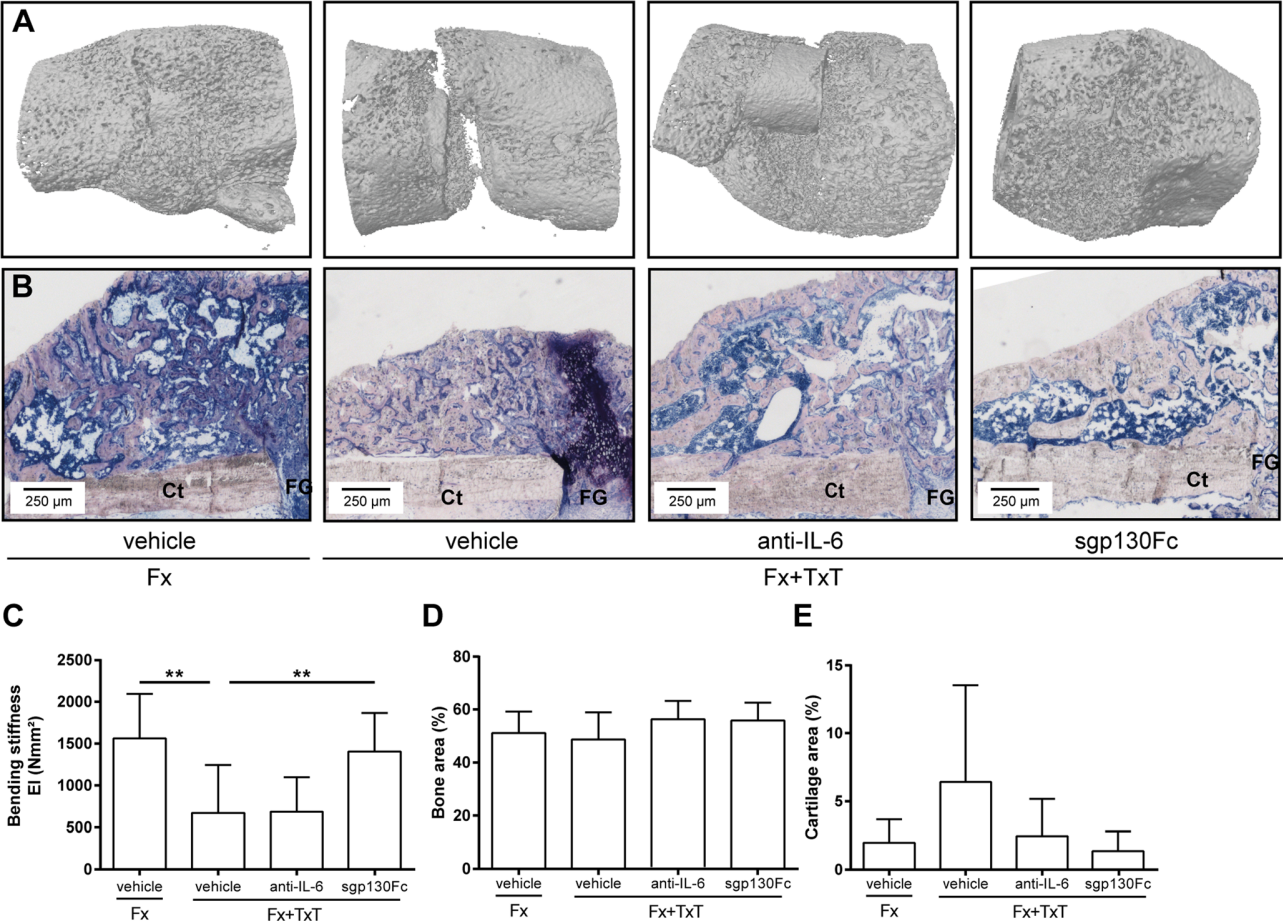
Micro-computer tomography, histomorphometrical, and biomechanical analyses of the fracture callus on day 21 after fracture (Fx) and combined fracture and thoracic trauma (Fx + TxT) in vehicle-, anti-IL-6 antibody-, and sgp130Fc-treated mice. **a** Representative μCT three-dimensional reconstructions of the fracture callus. **b** Representative Giemsa-stained histological images of the fracture callus. **c** Bending stiffness of fractured femurs. Relative amount of **d** bone and **e** cartilage determined by histomorphometrical analyses. Data are displayed as mean ± standard deviation. *n* = 7–9 (**c–e**). **p ≤ 0.01

Confirming our previous studies (Bergdolt et al. 2017; Kemmler et al. 2015; Kovtun et al. 2016), we found that the combined fracture and thoracic trauma (Fx + TxT) induced systemic and pulmonary inflammation and disturbed fracture healing. The plasma levels of IL-6, MCP-1, and CXCL1 were significantly increased 3 h after combined trauma compared to the isolated fracture (Fx) group indicating a systemic immune response post trauma (Fig. 1a, c, d). The sIL-6R concentration was significantly elevated in the combined trauma group at day 1 suggesting increased shedding of the mIL6R (Fig. 1b). All other measured circulating inflammatory mediators were not significantly affected compared to mice with isolated fracture. Furthermore, the combined trauma slightly increased *Crp* and *Saa* expression in the liver (Fig. 2a, b). In the lung, the combined trauma caused tissue damage and inflammation as confirmed by the presence of blood clots, alveolar wall thickening (Fig. 3a, b), increased neutrophil numbers (Fig. 3c, d), and elevated IL-6 and CXCL1 levels (Fig. 3e, f). In the fracture hematoma, the measured inflammatory mediators and immune cell recruitment to the fracture site were not significantly affected by the additional thoracic trauma (Fig. 4). However, fracture healing was disturbed by the trauma as indicated by a reduced bone fraction in the developing callus at day 10 (Fig. 5a, c), poor bony bridging of the fracture gap (Fig. 6a, b) and decreased mechanical properties of the fractured bone (Fig. 6c) in the late healing phase at day 21 compared to mice with isolated fracture. The relative amounts of bone and cartilage were not significantly altered at day 21 (Fig. 6d, e).

To block IL-6 signaling globally during the inflammatory phase, we administered an anti-IL-6 antibody 30 min and 2 days after combined trauma. The anti-IL-6 antibody considerably reduced IL-6 plasma levels compared to vehicle-treated mice 3 h after injury indicating efficient IL-6 inhibition. IL-6 plasma levels also remained low after 1 day (Fig. 1a). The trauma-induced increase of circulating sIL-6R was also significantly diminished by the anti-IL-6 antibody, suggesting that the shedding of the mIL-6R may be mediated by IL-6 (Fig. 1b). Whereas the trauma-induced increase of MCP-1 was not significantly affected after global IL-6 inhibition, CXCL1 and CRP plasma levels were significantly reduced 3 h and 1 day after combined injury, respectively (Fig. 1c–e). The hepatic acute-phase response was strongly diminished by the anti-IL-6 antibody treatment as indicated by significantly reduced *Crp*, *Saa*, and *Cxcl1* gene expression (Fig. 2a–c). Lung damage (data not shown) and neutrophil invasion into the lung tissue (Fig. 3c, d) after combined injury were not influenced by global IL-6 inhibition; however, IL-6 in the BAL fluid was significantly reduced compared to vehicle-treated mice (Fig. 3e).

In the fracture hematoma, IL-6 was also significantly reduced after global IL-6 inhibition compared to vehicle-treated mice (Fig. 4a); however, other measured inflammatory mediators were unaffected. FACS analysis revealed a reduced recruitment of neutrophils to the fracture site in mice with combined fracture and thoracic trauma that received the anti-IL-6 antibody (Fig. 4d). Histological and μCT evaluation demonstrated that the amount of newly formed bone and cartilage in the fracture callus were unaffected in the combined trauma group after global IL-6 inhibition at days 10 (Fig. 5) and 21 (Fig. 6). Additionally, the mechanical properties of the fracture callus were not significantly influenced (Fig. 6c).

In summary, global IL-6 inhibition reduced circulating IL-6, sIL-6R, and CXCL1, the hepatic acute-phase response, and neutrophil numbers in the fracture hematoma but did not influence the healing outcome after severe trauma.

### Inhibition of IL-6 trans-signaling improves compromised bone repair induced by combined fracture and thoracic trauma

To selectively block IL-6 trans-signaling, we treated mice with combined fracture and thoracic trauma with the artificial fusion protein sgp130Fc in the early posttraumatic phase 30 min and 2 days after injury. IL-6 trans-signaling inhibition significantly reduced circulating IL-6 3 h after combined trauma, but significantly increased it after 1 day compared to vehicle-treated mice (Fig. 1a). The trauma-induced increase of sIL-6R observed after 1 day was also significantly diminished (Fig. 1b). Other systemic inflammatory mediators were not significantly affected by sgp130Fc administration compared to vehicle-treated mice. In the liver, expression of *Crp* was significantly diminished after IL-6 trans-signaling inhibition (Fig. 2a). *Saa* and *Cxcl1* expression were also slightly reduced, although not significantly (Fig. 2b, c). In the lungs, sgp130Fc treatment affected neither lung damage (data not shown) nor the inflammatory response induced by the thoracic trauma (Fig. 3). Additionally, the early inflammation at the fracture site was not significantly influenced after IL-6 trans-signaling inhibition (Fig. 4). The bone and cartilage fractions in the developing fracture callus were unaltered after the blockade of IL-6 trans-signaling (Fig. 5a, c, d). However, the amount of collagen type X expressing hypertrophic cartilage was significantly increased indicating accelerated cartilage-to-bone transformation (Fig. 5b, e). Confirming this, 21 days after trauma, bony bridging of the fracture gap (Fig. 6a, b) and the bending stiffness (Fig. 6c) of the fracture callus were significantly elevated in mice treated with sgp130Fc, suggesting that the selective blockade of IL-6 trans-signaling significantly improved the fracture healing outcome after severe trauma.

## Discussion

**Fig. 7:**
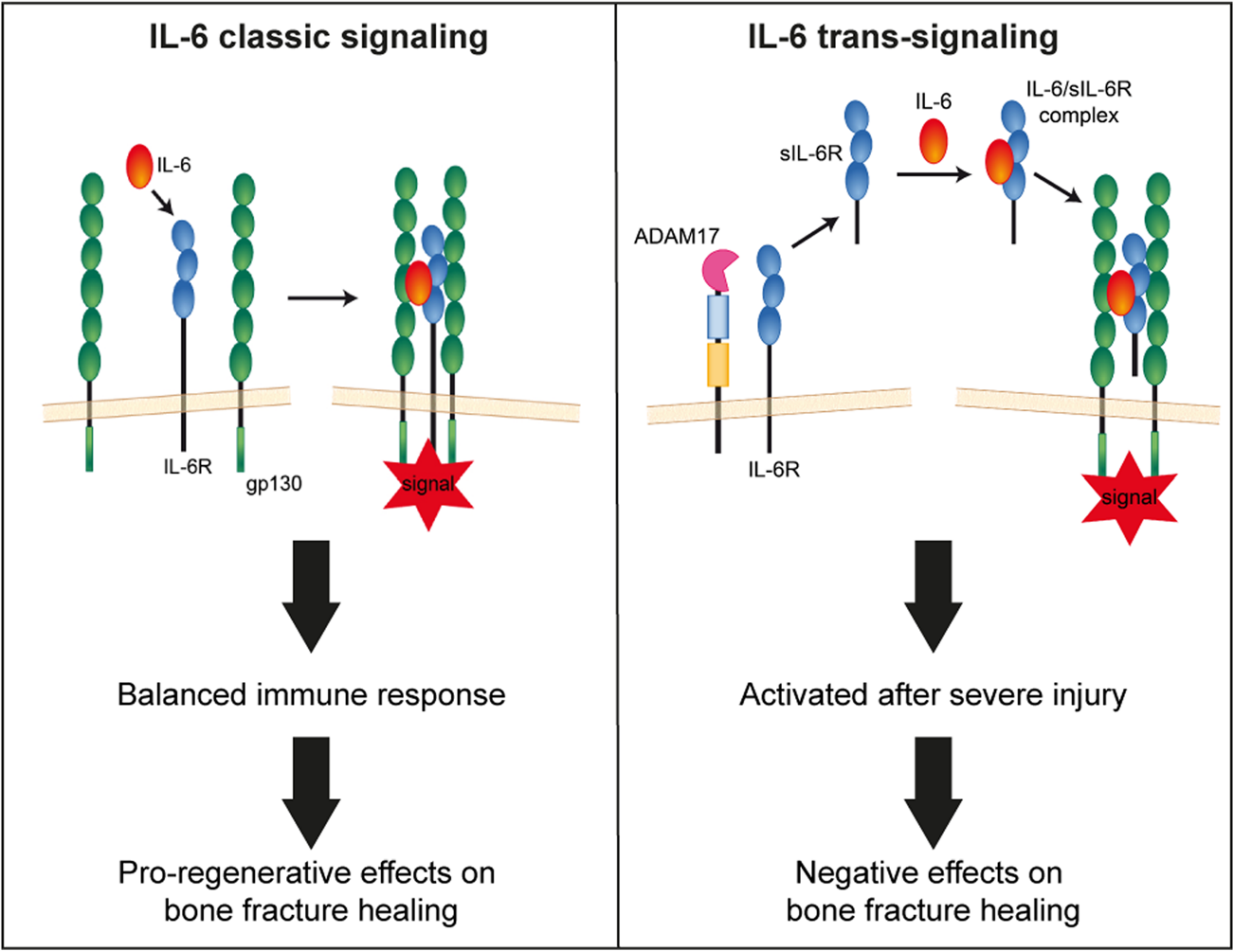
Scheme of IL-6 classic and trans-signaling and the proposed effects of fracture healing. In IL-6 classic signaling, IL-6 binds to its membrane-bound receptor (IL-6R), which then binds to a dimer of transmembrane glycoprotein 130 (gp130), inducing intracellular signal transduction. In IL-6 trans-signaling, IL-6 binds to its soluble receptor (sIL-6R), which is mainly shed by A Disintegrin and Metalloproteinase 17 (ADAM 17). The IL-6/sIL-6r complex then binds to the gp130 dimer. Our previous (Prystaz et al. 2017) and present results indicate that IL-6 classic signaling induces a balanced immune response and pro-regenerative effects on bone repair. In contrast, IL-6 trans-signaling, which is induced after severe injury, negatively affects fracture healing

Here, we investigated the hypothesis that IL-6 trans-signaling is involved in the pathomechanisms of trauma-induced compromised fracture healing. Using a mouse model of severe injury, we demonstrated that the transient blockade of IL-6 trans-signaling in the early posttraumatic phase with sgp130Fc significantly improved bone repair. By contrast, healing was not improved by an anti-IL-6 antibody, which blocks both IL-6 classic and trans-signaling, suggesting that the classic pathway rather exerts beneficial effects of augmenting bone repair under conditions of severe trauma, as it similarly does in uncomplicated fracture healing (Prystaz et al. 2017) (Fig. 7).

### IL-6 classic and trans-signaling differently modulate systemic posttraumatic inflammation

In this study, we used a mouse model of combined fracture and thoracic trauma to elucidate the role of IL-6 in compromised fracture healing after severe injury. As expected (Bergdolt et al. 2017; Kemmler et al. 2015; Kovtun et al. 2016), the combined injury induced a systemic inflammation with increased plasma levels of inflammatory mediators, including IL-6, and sIL-6R, which indicated enhanced shedding of the mIL-6R. This is in agreement with experimental (Kleber et al. 2015) and clinical studies (Beeton et al. 2004) in subjects with fracture and concomitant injury, and confirms that IL-6 trans-signaling is activated in posttraumatic inflammation.

To discriminate between IL-6 actions, we applied an anti-IL-6 antibody, which inhibits IL-6 globally, and sgp130Fc, which selectively blocks IL-6 trans-signaling (Jostock et al. 2001). It is not possible to inhibit IL-6 classic signaling selectively. However, by comparing the effects of global and trans-signaling inhibition, indirect, but valid conclusions can be drawn about the role of IL-6 classic signaling (Barkhausen et al. 2011; Prystaz et al. 2017). The anti-IL-6 antibody efficiently reduced IL-6 levels in the blood and BAL fluid. Global IL-6 inhibition also decreased serum levels of the sIL-6R, suggesting that IL-6 directly and indirectly stimulates the shedding of its membrane-bound receptor during posttraumatic inflammation (Lokau et al. 2017). While sgp130Fc diminished the trauma-induced increase of circulating IL-6 less efficiently after 3 h, it notably enhanced it after 1 day. The initial reduction of IL-6 may result from trapping of the IL-6/sIL-6R complex by sgp130Fc, which favors the restoration of the initial equilibrium of IL-6/sIL-6R complexes and, thus, reduces circulating free IL-6 molecules (Garbers et al. 2011). The later increase of IL-6 may be caused by a delay of IL-6 degradation after interception of IL6/sIL-6R complexes by sgp130Fc and was also found in patients, who were treated with the anti-IL-6R antibody tocilizumab (Nishimoto et al. 2008).

The anti-IL-6 antibody also abolished the trauma-induced increase of CXCL1 in the circulation and its hepatic expression. By contrast, selective inhibition of IL-6 trans-signaling did not significantly reduce circulating CXCL1. This is in agreement with our previous data (Prystaz et al. 2017) indicating that the liver is a major source of CXCL1 after injury, and that hepatic CXCL1 production is regulated by IL-6 classic signaling. The classic IL-6 pathway is known to induce the hepatic acute-phase response (Schmidt-Arras and Rose-John 2016). Confirming this, the posttraumatic expression of CRP and SAA in the liver was significantly reduced by the anti-IL-6 antibody, whereas inhibition of IL-6 trans-signaling provoked only minor effects. Hepatic IL-6 classic signaling is suggested to act pro-regenerative, as it induces the first-line defense against pathogens and limits inflammatory responses (Schmidt-Arras and Rose-John 2016).

### IL-6 classic and trans-signaling differently modulate inflammation at the site of fracture

In the fracture hematoma, the anti-IL-6 antibody reduced IL-6 as expected, but neither global nor trans-signaling inhibition affected the concentration of other inflammatory mediators. The proportion of neutrophils was significantly reduced after global but not after trans-signaling inhibition, indicating that IL-6 classic signaling regulates neutrophil recruitment and/or apoptosis directly or indirectly. This corroborates our previous study in the isolated fracture model (Prystaz et al. 2017) and can be explained by the reduced plasma concentrations of the neutrophil chemoattractant CXCL1. Studies about direct IL-6 effects on neutrophil functions are conflicting. In an air-pouch model, neutrophil trafficking was induced by IL-6 trans-signaling (Rabe et al. 2008), whereas in a mouse model of peritoneal inflammation, IL-6-induced STAT3-signaling diminished neutrophil recruitment (Fielding et al. 2008). Others reported that IL-6 did not directly act as a neutrophil chemoattractant or induce apoptosis (Wright et al. 2014), although its therapeutic inhibition by tocilizumab induces neutropenia (Espinoza et al. 2017; Wright et al. 2014). The proportions of other immune cell populations in the fracture hematoma were not affected by global or trans-signaling inhibition. This is in contrast to the isolated fracture model, where monocytes, macrophages, and lymphocytes were significantly reduced after IL-6 inhibition (Prystaz et al. 2017). The reason for the different results in the isolated fracture and combined fracture and thoracic trauma models could be that severe trauma affects the phenotype and function of many immune cells (Flohe et al. 2008; Lord et al. 2014), possibly also their responsiveness to IL-6.

### Inhibition of trans-signaling, but not global IL-6 inhibition, ameliorates the deleterious effects of a concomitant injury on bone repair

Our study demonstrated that global IL-6 inhibition did not affect trauma-induced impaired fracture healing. By contrast, inhibition of IL-6 trans-signaling accelerated cartilage-to-bone transformation in the intermediate healing phase, and enhanced bony bridging of the fracture gap and mechanical callus properties in the late stage. This suggests that, under conditions of severe trauma, IL-6 trans-signaling mediates negative effects on bone repair, whereas classic signaling may act rather pro-regenerative, as it similarly does in uncomplicated fracture healing (Prystaz et al. 2017). But how can the positive effects of IL-6 trans-signaling inhibition be explained mechanistically? One difference between both treatment groups was the reduced neutrophil number in the fracture hematoma after global but not after trans-signaling inhibition. Neutrophils are the most abundant immune cell population in the early fracture hematoma (Hoff et al. 2016). They remove pathogens, coordinate the transition to a more sustained population of mononuclear cells, and contribute to the resolution of inflammation (Bastian et al. 2011; Kovtun et al. 2016). Neutrophils may augment bone regeneration, because it is impaired after neutrophil depletion (Chan et al. 2015; Kovtun et al. 2016). However, after severe trauma, neutrophils can become dysfunctional and aggravate tissue damage, for example, by the massive production of reactive oxygen species (ROS) and neutrophil extracellular traps (NETs) (Hazeldine et al. 2014). Therefore, the role of neutrophils in fracture healing might depend on trauma severity. A limitation of the present study is that we did not assess neutrophil activity at the fracture site. Therefore, their role remains unclear. Further work is necessary to elucidate neutrophil functions in fracture healing and how they are regulated by IL-6. Another striking observation, which could possibly explain improved bone repair was the altered kinetics of circulating IL-6 in sgp130Fc-treated mice. After initial reduction, IL-6 plasma levels were moderately, but significantly increased after 1 day, whereas they remained low after anti-IL-6 antibody treatment. As explained above, this could result from delayed IL-6 degradation after sgp130Fc treatment (Rose-John et al. 2007). Possibly, the free IL-6 then provokes rather pro-regenerative effects by activating classic signaling, because trans-signaling might still be inhibited. However, a limitation of the present study is that we did not include enough early investigation time points to unravel the interconnection between the early immune response and regenerative processes. Further investigations are needed to mechanistically explain improved bone regeneration in trauma-induced compromised fracture healing by sgp130Fc.

## Conclusions

In summary, the present study demonstrates for the first time that IL-6 trans-signaling is involved in the pathomechanisms of compromised fracture healing after severe injury, whereas IL-6 classic signaling rather mediates pro-regenerative effects augmenting bone regeneration. However, further studies are necessary to elucidate the underlying mechanisms in detail. Nevertheless, our results can help to develop new treatment strategies to reduce fracture healing complications after severe injury.

## Electronic supplementary material

Supplemental Table 1 Inflammatory mediators in the blood and broncho-alveolar lavage (BAL) fluid of untreated mice. Data are presented as the means ± standard deviation. *n* = 4. #, below the detection limit of the used assay. IL-6, interleukin-6; sIL-6R, soluble IL-6 receptor; IFNγ, interferon-γ; IL-1β, interleukin-1β; TNF-α, tumor necrosis factor-α; IL-10, interleukin-10; IL-13, interleukin-13; IL-4, interleukin-4; CXCL-1, chemokine (C-X-C motif) ligand 1; MCP-1, monocyte chemotactic protein 1; MIP-1α, macrophage inflammatory protein-1α; CRP, C-reactive protein (GIF 47 kb)

High resolution image (TIFF 3327 kb)
