# Supplementary figures and images for: Pharmacological inhibition of IL-6 trans-signaling improves compromised fracture healing after severe trauma
Source: Naunyn Schmiedebergs Arch Pharmacol. 2018 Mar 1;391(5):523–36. doi: 10.1007/s00210-018-1483-7 (PMC5889421; doi:10.1007/s00210-018-1483-7)

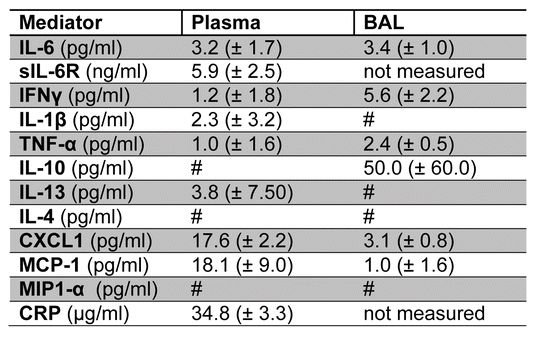

Supplement: Supplementary file 1 — Inflammatory mediators in the blood and broncho-alveolar lavage (BAL) fluid of untreated mice. Data are presented as the means ± standard deviation. n = 4. #, below the detection limit of the used assay. IL-6, interleukin-6; sIL-6R, soluble IL-6 receptor; IFNγ, interferon-γ; IL-1β, interleukin-1β; TNF-α, tumor necrosis factor-α; IL-10, interleukin-10; IL-13, interleukin-13; IL-4, interleukin-4; CXCL-1, chemokine (C-X-C motif) ligand 1; MCP-1, monocyte chemotactic protein 1; MIP-1α, macrophage inflammatory protein-1α; CRP, C-reactive protein (GIF 47 kb) [file 210_2018_1483_Fig8_ESM.gif]

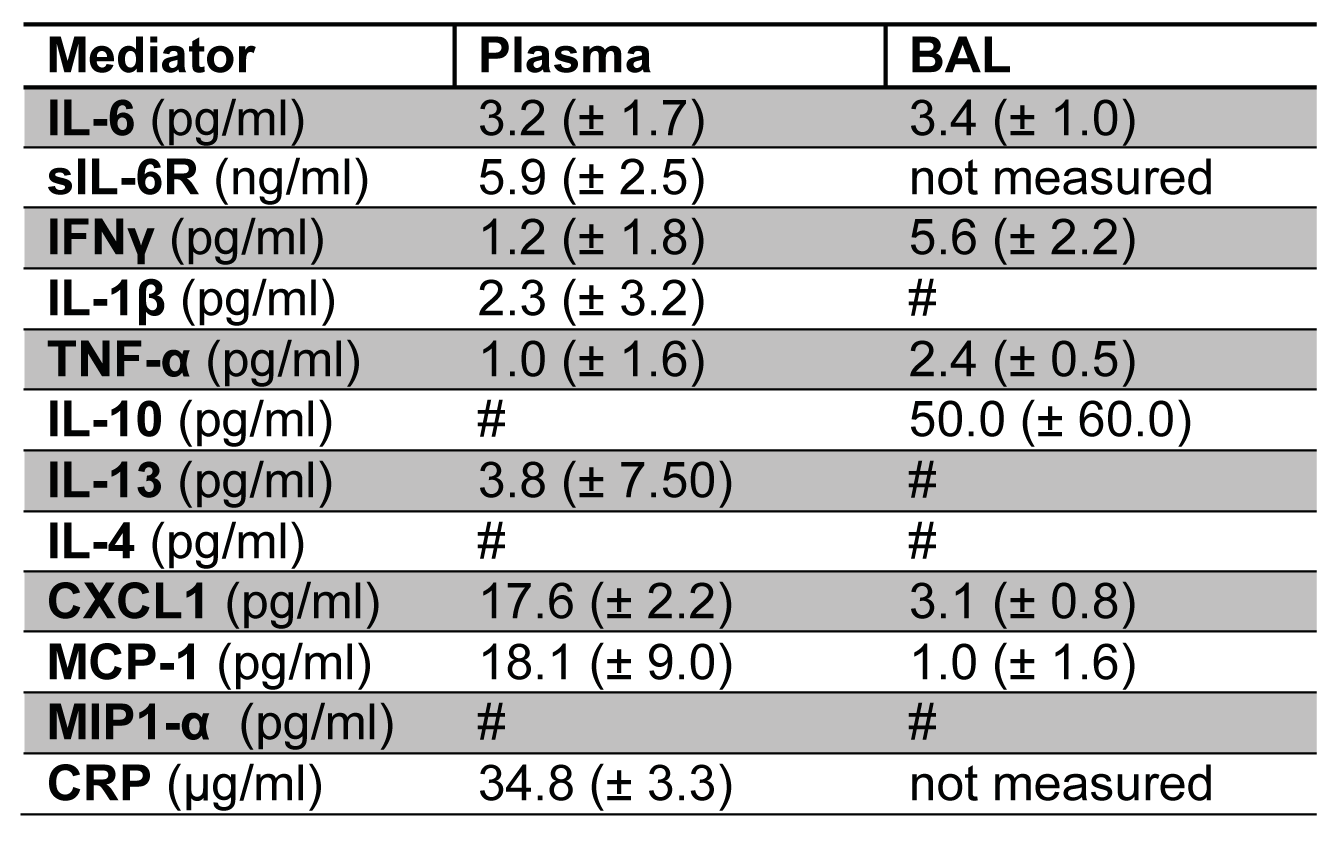

Supplement: Supplementary file 2 — High resolution image (TIFF 3327 kb) [file 210_2018_1483_MOESM1_ESM.tif]
